# Additional description and genome analyses of *Caenorhabditis auriculariae* representing the basal lineage of genus *Caenorhabditis*

**DOI:** 10.1038/s41598-021-85967-z

**Published:** 2021-03-24

**Authors:** Mehmet Dayi, Natsumi Kanzaki, Simo Sun, Tatsuya Ide, Ryusei Tanaka, Hayato Masuya, Kimiko Okabe, Hisashi Kajimura, Taisei Kikuchi

**Affiliations:** 1 Department of Infectious Disease, Faculty of Medicine, University of Miyazaki, 5200 Kiyotakecho Kihara, Miyazaki-City, Miyazaki 889-1692 Japan; 2 Forestry Vocational School, Duzce University, 81620 Duzce, Turkey; 3 Kansai Research Center, Forestry and Forest Products Research Institute, 68 Nagaikyutaroh, Momoyama, Fushimi, Kyoto 612-0855 Japan; 4 Department of Zoology, National Museum of Nature and Science, 4-1-1 Amakubo, Tsukuba, Ibaraki 305-0005 Japan; 5 Forestry and Forest Products Research Institute, 1 Matsunosato, Tsukuba, Ibaraki 305-8687 Japan; 6 Graduate School of Bioagricultural Sciences, Nagoya University, Furo-cho, Chikusa-ku, Nagoya, Aichi 464-8601 Japan

**Keywords:** Evolution, Taxonomy, Genomics

## Abstract

*Caenorhabditis auriculariae,* which was morphologically described in 1999, was re-isolated from a *Platydema* mushroom-associated beetle. Based on the re-isolated materials, some morphological characteristics were re-examined and ascribed to the species. In addition, to clarify phylogenetic relationships with other *Caenorhabditis* species and biological features of the nematode, the whole genome was sequenced and assembled into 109.5 Mb with 16,279 predicted protein-coding genes. Molecular phylogenetic analyses based on ribosomal RNA and 269 single-copy genes revealed the species is closely related to *C. sonorae* and *C. monodelphis* placing them at the most basal clade of the genus. *C. auriculariae* has morphological characteristics clearly differed from those two species and harbours a number of species-specific gene families, indicating its usefulness as a new outgroup species for *Caenorhabditis* evolutionary studies. A comparison of carbohydrate-active enzyme (CAZy) repertoires in genomes, which we found useful to speculate about the lifestyle of *Caenorhabditis* nematodes, suggested that *C. auriculariae* likely has a life-cycle with tight-association with insects.

## Introduction

The nematode, *Caenorhabditis elegans,* has been a core model organism that is used in a wide range of biological and medical studies for the last several decades and has led to a number of key discoveries, including the molecular mechanisms of apoptosis^1^ and gene silencing by small RNAs^2^. Recently, *C. elegans* and other species of the genus *Caenorhabditis* have been developed as a useful model system for a wide range of evolutionary studies^3,4^.

Currently, the genus *Caenorhabditis* contains 49 nominal species^5–8^. However, because of the morphological similarity among close relatives and morphological divergence within each species, the species status is often delimited on molecular barcoding and/or hybridization analyses^4^, and several species have been described only based on molecular phylogenetic status and mating studies^6^. Meanwhile, some previously described species, including *C. anthobia*, *C. avicola*, *C. clavopapillata,* and *C. craspedocercus,* were characterized based solely on their morphological traits (Table 1, Supplementary Text). For those species, re-isolation followed by molecular profiling and/or re-characterization of typological characteristics is demanded.

**Table 1:** List of nominal and characterized *Caenorhabditis* species including type locality, and type host or habitat.

Species name with taxonomic author(s)	Type locality	Type host or habitat	Previous species code (type strain)	Reference (original description)
*C. afra* Félix, Braendle & Cutter, 2014	Begoro, Ghana	Rotting citrus fruit	sp. 7 (JU1199)	Félix et al. (2014) PLoS ONE 9: e94723
*C. angaria* Sudhaus, Kiontke & Giblin-Davis, 2011	Frog Pond, Dade County, Florida, USA	Sugarcame weevil *Metamasius hemipterus* (Olivier) (Curculionidae)	sp. 3 (PS1010)	Sudhaus et al. (2011) Nematology 13: 61–78
*C. anthobia* (W. Schneider, 1937)	South-Sumatra	Water in the inflorescences of *Cyrtandra glabra* (Gesneriaceae)		Schneider (1937) Arch Hydrobiol (Suppl.) 15: 30–108
*C. auriculariae* Tsuda & Futai, 1999	North Canpus of Kyoto University, Kyoto, Japan	Fruiting bodies of the cloud ear fungus *Auricularia polytricha* (Montagne) (Auricularaceae), generated on a trunk of the red elder *Sambucus racemosa* L. (Adoxaceae)		Tsuda & Futai (1999) Jpn J Nematol 29: 18–23
*C. avicola* Schmidt & Kuntz, 1972	Chien-shih, Hsinchu Hsien, Taiwan	Small intestine of a plumbeous water redstart *Rhyacornis fuliginosus affinis* Vigors (Muscicapidae)		Schmidt & Kuntz (1972) Proc Helminthol Soc Wash 39: 189–191
*C. becei* Stevens & Félix, 2019	Barro Colorado Island, Panama	Rotting flower of *Gustavia superba*	sp. 29 (QG704)	Stevens et al. (2019) Evolution Letters 3: 217–236
*C. bovis* (Kreis, 1964)	Mkwaya-Ranch of Amboni Est. Ltd., coastal area of Tanzania (East Africa)	Outer auditory canal of male zebu, *Bos* sp. (Bovidae)		Kreis (1964) Schweiz Arch Tierheilkd 106: 372–378
*C. brenneri* Sudhaus & Kiontke, 2007	Bohorok, Sumatra	Compost-like material, mostly banana leaves	sp. 4 (SB129)	Sudhaus & Kiontke (2007) Zootaxa 1456: 45–62
*C. briggsae* (Dougherty & Nigon, 1949)	Campus of Stanford University, Palo Alto, California, USA	Soil		Dougherty & Nigon (1949) J Parasitol (Suppl.) 35: 11
*C. castelli* Félix, Braendle & Cutter, 2014	“Petit Plateau” near the CNRS Biological station, Nouragues, French Guiana	Rotting *Micropholis cayennensis* fruit	sp. 12 (JU1426)	Félix et al. (2014) PLoS ONE 9: e94723
*C. chinkari* Mondal & Manna, 2015	Alipore Zoological Garden, Kolkata, West Bengal, India	Faeces of captive chinkara, *Gazella gazella bennettii* (Sykes) (Bovidae)		Mondal & Manna (2015) Proc Zool Soc (Kolkata) 68: 52–57
*C. clavopapillata* (Kreis & Faust, 1933)	New Orleans, Louisiana, USA	Faeces-soiled hair in the perianal region of a dog		Kreis & Faust (1933) Trans Amer Microscop Soc 52: 162–172
*C. craspedocercus* (Völk, 1950)	Erlangen, Germany	Earthworm, *Eisenia rosea* (Savigny) (Lumbricidae)		Völk (1950) Zool Jahrb (Systematik) 79: 1–70
*C. doughertyi* Félix, Braendle & Cutter, 2014	Angela Spice Garden a few km from Periyar, Kerala, India	Rotting cacao fruit	sp. 10 (JU1333)	Félix et al. (2014) PLoS ONE 9: e94723
*C. drosophilae* (Kiontke, 1997)	Pima County, Tucson Mountains, Arizona, USA	Decaying tissue of saguaro cactus, *Carnegiea gigantea* (Engelm.) (Cactaceae); dauer juveniles phoretic on *Drosophila nigrospiracula* Patterson and Wheeler (Drosophilidae)		Kiontke (1997) Fundam Appl Nematol 20: 305–315
*C. elegans* (Maupas, 1899)	Surroundings of Algiers, Algeria	Rich humus		Maupas (1899) Arch Zool Exp Gen 7: 563–628
*C. formosana* (Yokoo & Okabe, 1968)	Puyen Village, Changhua County, Taiwan	Amphibious snail *Oncomelania hupensis formosana* Gredler (Rissooidea)		Yokoo & Okabe (1968) Agric Bull Saga Univ 25: 69–78
*C. fruticicolae* (Shinohara, 1960)	Monkara, Miyazaki, Japan	Intestine of the snail *Fruticicola (Acusta) sieboldiana* (Pfeiffer) (Helicoidea)		Shinohara (1960) J Kurume Med Assoc 23: 2777–2819
*C. genitalis* (Scheiber, 1880)	Stuhlweissenburg, Hungary	Outer genitals of a bedfast woman		Scheiber (1880) Arch Pathol Anat Physiol Klin Med 82: 161–175
*C. guadeloupensis* Félix, Braendle & Cutter, 2014	Soufrière, Forest trail, Guadeloupe, France (Overseas department)	Rotten *Heliconia* flowers	sp. 20 (NIC113)	Félix et al. (2014) PLoS ONE 9: e94723
*C. imperialis* Félix, Braendle & Cutter, 2014	Moorea, French Polynesia	Rotting horse chestnut	sp. 14 (EG5716)	Félix et al. (2014) PLoS ONE 9: e94723
*C. inopinata* Kanzaki, Tsai, Tanaka, Hunt, Liu, Tsuyama, Maeda, Namai, Kumagai, Tracey, Holroyd, Doyle, Woodruff, Murase, Kitazume, Chai, Akagi, Panda, Ke, Schroeder, Wang, Berriman, Sternberg, Sugimoto & Kikuchi, 2018	Ishigaki Isl., Okinawa, Japan	Fresh syconia of *Ficus septica*	sp. 34	Kanzaki et al. (2018) Nature Communications 9: 3216
*C. japonica* Kiontke, Hironaka & Sudhaus, 2003	Hinokuma Mountain Prefectural Park, Kanzaki Town, Saga, Japan	Dauer juveniles found on females of the shield bug *Parastrachia japonensis* Scott (Heteroptera)		Kiontke et al. (2003) Nematology 4 (2002): 933–941
*C. kamaaina* Félix, Braendle & Cutter, 2014	Kaui, Hawaii, USA	Unidentified wild rotten fruit	sp. 15 (QG122)	Félix et al. (2014) PLoS ONE 9: e94723
*C. latens* Félix, Braendle & Cutter, 2014	Juifeng Village, Wuhan City, Hubei Province, China	Soil near the lotus pond	sp. 23 (VX88)	Félix et al. (2014)PLoS ONE 9: e94723
*C. macrosperma* Félix, Braendle & Cutter, 2014	Nouragues Forest (path to Inselberg), French Guiana	Rotten *Hyeronima taxiflora* fruits (nut)	sp. 18 (JU1857)	Félix et al. (2014) PLoS ONE 9: e94723
*C. monodelphis* Slos & Sudhaus, 2017	Berlin, Germany	Galleries inside of the fungus *Ganoderma applanatum* (Polyporaceae) which grew on the stump of a tree a few centimeters above ground	sp. 1 (SB341)	Slos et al. (2017) BMC Zool 2: 4
*C. nigoni* Félix, Braendle & Cutter, 2014	Zoo/Botanical Garden of Trivandrum, Kerala, India	Rotting flowers and leaves	sp. 9 (JU1325)	Félix et al. (2014) PLoS ONE 9: e94723
*C. nouraguensis* Félix, Braendle & Cutter, 2014	Grand Plateau in Nouragues Forest, French Guiana	Rotten wide bean of *Barokia* sp.	sp. 17 (JU1825)	Félix et al. (2014) PLoS ONE 9: e94723
*C. oncomelaniae* (Yokoo & Okabe, 1968)	Kurume, Japan (isolated from lavoratory-rared population of host, but the origin of the population is not specified in the description)	Snail, *Oncomelania hupensis nosophora* Gredler (Rissooidea)		Yokoo & Okabe (1968) Agric Bull Saga Univ 25: 69–78
*C. panamensis* Stevens & Félix, 2019	Barro Colorado Island, Panama	Rotting palm fruit	sp. 21 (QG702)	Stevens et al. (2019) Evolution Letters 3: 217–236
*C. parvicauda* Stevens & Félix, 2019	Sainte Marie Island, Madagascar	Apple-like rotten and dry fruit	sp. 21 (NIC206)	Stevens et al. (2019) Evolution Letters 3: 217–236
*C. plicata* (Völk, 1950)	Erlangen, Germany	*Hister stercorarius* Hoffmann (Histeridae) from carrion; phoretic on different carrion beetles (Silphidae)		Völk (1950) Zool Jahrb (Systematik) 79: 1–70
*C. perrieri* (Maupas, 1900)	Near Mitidja, Arba, Algeria	Soil at the bottom of a manure heap		Maupas (1900) Arch Zool Exp Gen 8: 463–624
*C. portoensis* Félix, Braendle & Cutter, 2014	Amares, Portugal	Rotting apple	sp. 6 (EG4788)	Félix et al. (2014) PLoS ONE 9: e94723
*C. quiockensis* Stevens & Félix, 2019	Basse-Terre Forest, Guadeloupe	Rotting fruit (species not specified)	sp. 38 (JU2745)	Stevens et al. (2019) Evolution Letters 3: 217–236
*C. remanei* (Sudhaus, 1974)	Freiburg, Germany	Slug *Arion rufus* L. (Arionidae)		Sudhaus (1974) Zool Jahrb (Systematik) 101: 417–465 Baird et al. (1994) Nematologica 40: 1–11
*C. sinica* Huang, Ren, Qiu & Zhao, 2014	Luofu Mountain of Huizhou City, Guangdong Province, China	Rotting fruit (species not specified)	sp. 5 ZZY0401	Huang et al. (2014) PLoS ONE 9: e110957
*C. sonorae* (Kiontke, 1997)	Pima County, Tucson Mountains, Arizona, USA	Decaying tissue of saguaro cactus, *Carnegiea gigantea* (Engelm.) (Cactaceae)		Kiontke (1997) Fundam Appl Nematol 20: 305–315
*C. sulstoni* Stevens & Félix, 2019	East Africa	Faeces of millipede *Archispirostreptus gigas* bought in spring 2013 on an insect market in Berlin	sp. 32 (SB454)	Stevens et al. (2019) Evolution Letters 3: 217–236
*C. tribulationis* Stevens & Félix, 2019	Danbulla, Australia	Humus below *Ficus destruens*	sp. 40 (JU2774 )	Stevens et al. (2019) Evolution Letters 3: 217–236
*C. tropicalis* Félix, Braendle & Cutter, 2014	La Réunion, France (Overseas department)	Rotting torch ginger (rose porcelaine—*Etlingeria elatior*) flowers	sp. 11 (JU1373)	Félix et al. (2014) PLoS ONE 9: e94723
*C. uteleia* Stevens & Félix, 2019	Madre de Dios, Peru	Rotting Kumquat-like fruit	sp. 31 (JU2469)	Stevens et al. (2019) Evolution Letters 3: 217–236
*C. virilis* Félix, Braendle & Cutter, 2014	Orsay, France	Rotting apple	sp. 13 (JU1528)	Félix et al. (2014) PLoS ONE 9: e94723
*C. vivipara* Stevens & Félix, 2019	Chichén Itzá, Yucatán, Mexico	Rotting red berries	sp. 43 (NIC1070 )	Stevens et al. (2019) Evolution Letters 3: 217–236
*C. wallacei* Félix, Braendle & Cutter, 2014	Cacao plantation near Sanda Center, Bali, Indonesia	Rotten cacao fruit	sp. 16 (JU1873)	Félix et al. (2014) PLoS ONE 9: e94723
*C. waitukubuli* Stevens & Félix, 2019	Morne Trois Pitons Trail, Forest, Dominica	Rotting *Clusia* fruit	sp. 39 (NIC564)	Stevens et al. (2019) Evolution Letters 3: 217–236
*C. yunquensis* Félix, Braendle & Cutter, 2014	El Yunque, Puerto Rico	Not provided (rotten fruit?)	sp. 19 (EG6142)	Félix et al. (2014) PLoS ONE 9: e94723
*C. zanzibari* Stevens & Félix, 2019	Jozani, Zanzibar	Rotting fruit of *Calophyllum inophyllum*	sp. 26 (JU2161)	Stevens et al. (2019) Evolution Letters 3: 217–236
*Caenorhabditis* sp. 2	Mediterranean islands (Mallorca, Sardinia), Madeira, Portugal and the Canary Islands, Spain	Isolated from rotting *Opuntia* cactus tissue, and probably carried by *Drosphila* sp.		Kiontke et al. (2011) BMC Evol Biol 11: 339
*Caenorhabditis* sp. 8	New Jersey, USA	Rotting tomatoes		Kiontke et al. (2011) BMC Evol Biol 11: 339

*Caenorhabditis auriculariae* was initially described morphologically as an associate of the fruiting bodies of the basidiomycota fungus *Auricularia polytricha*^9^. Based on morphological characteristics, the species is relatively easily distinguished from other congeners^9^. For example, the species has a short stoma, bifurcated metastegostomatal teeth, a spicule structure similar to the *drosophilae* super group, and closed bursa similar to several species in the *drosophilae* and *elegans* super group species. Thus, this species seems to be rather basal in the genus, but the phylogenetic status of the species remained undetermined.

In the present study, using re-isolated materials, we examined *C. auriculariae* morphological characteristics in details, and defined the basic molecular profiles based on nuclear ribosomal sequences. Additionally, we sequenced the whole genome of the species and revealed its basic genomic features. These results revealed the *C. auriculariae*’s basal phylogenetic position in the genus and its usefulness as an outgroup to understand the *Caenorhabditis* evolution.

## Results

### Morphological characteristics

**Figure 1:**
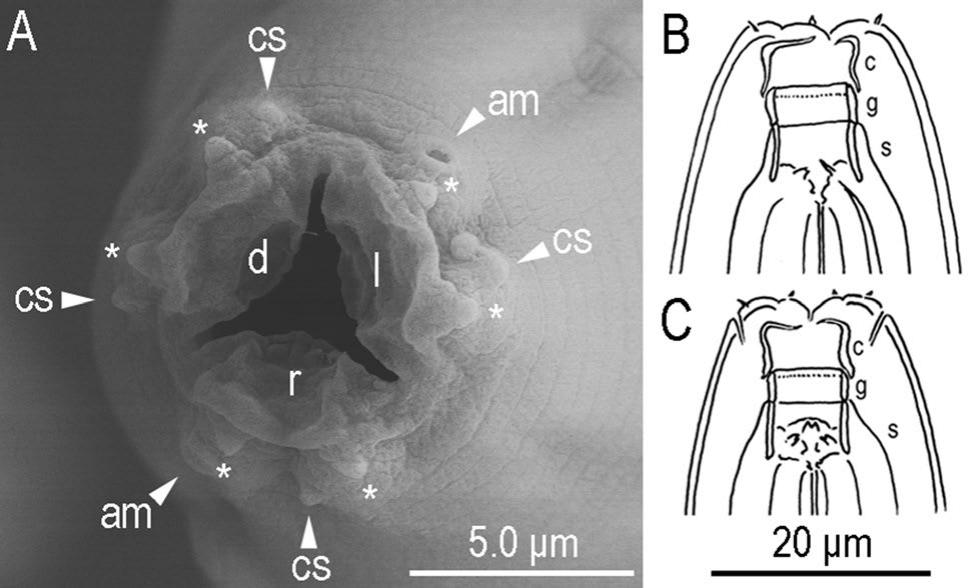
Stomatal structure of *Caenorhabditis auriculariae*. (**A**) En face view where lavial censilla (asterisk), cephalic sensilla (c), amphids (a) and cheilostomatal flap baring on dorsal (d), right subventral (r) and left subventral (l) sectors are suggested; (**B**) left lateral view; (**C**) ventral view. Cheilostom (c), gymnostom (g) and stegostom (s) are suggested in (**B**,**C**).

*Caenorhabditis auriculariae* is a rare, neglected species which was reported only once about 20 years ago^9^. We isolated *C. auriculariae* from a mushroom beetle, *Platydema* sp. feeding on the fruiting body of *Auricularia polytricha* in Aichi, Japan. The general morphological characteristics are as described previously^9^ and those are photo-documented and illustrated in Fig. 1 and Supplementary Figs. S1–S7. Several typological taxonomic/phylogenetic key characteristics in the male tail are explained and discussed below. Only newly found characteristics are described here to avoid redundancy.

The detailed lip and cheilostomatal structures are described for the first time as follows (Fig. 1). Lip is separated into six lip sectors, and each has an outer labial papilla. There, three pairs of neighboring (right subventral + latelal, left subventral + lateral and right and left dorsal) lip sectors are partially fused to form three large lip sectors. Thus, these three sectors are arranged triradially in *en face* view. The short tube-like stoma is separated into three elements from anterior: cheilostom, gymnostom and stegostom. Cheilostom cuticular tube, occupies about 35–40% of total stomatal length. Anterior part of cheilostomatal wall (cheilorhabdion) extends and internally fold to form a half-circle shaped flap which covers stomatal opening like valve apparatus. Posterior end of chaeilostom overlapping gymnostom. Gymnostom is simple and short cuticular tube, occupies about 20–25% of stoma. Stegostom is separated from the other parts of stomatal element by possession of pharyngeal sleeve consisting of four subelements: pro-, meso-, meta- and telostogostom. Pro- and mesostegostom is not clearly separated and forms a simple cuticular tube. Metastegostom at the posterior end of pro-mesostegostomatal tube, forming three (two subventral and a dorsal) small bifid teeth. Telostegostom not cuticularized, connecting stoma and pharynx.

### Phylogenetic status

**Figure 2:**
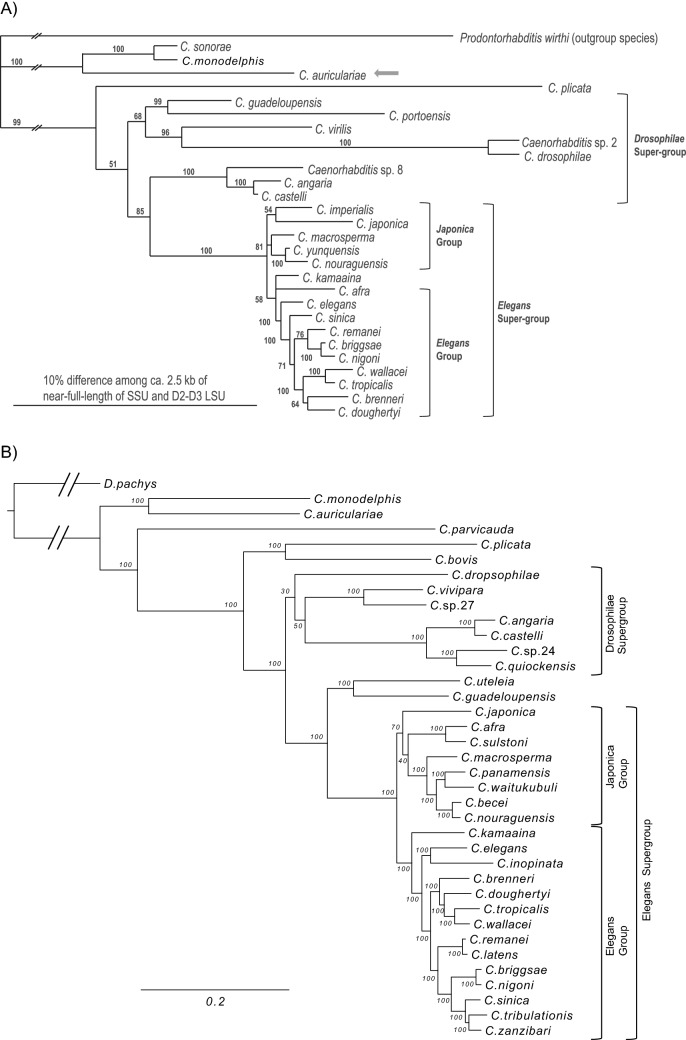
Phylogenetic status of *Caenorhabditis auriculariae*. (**A**) The combined Bayesian tree inferred from near-full-length SSU and D2-D3 LSU. The substitution model and parameters for SSU and D2-D3 are GTR + I + G (AIC = 16,046.5234; lnL = 8013.261; freq A = 0.2452; freq C = 0.2064; freq G = 0.2661; freq T = 0.2823; R(a) = 1.4800; R(b) = 3.4097; R(c) = 1.9899; R(d) = 0.5864; R(e) = 5.6910; R(f) = 1; Pinva = 0.4188; Shape = 0.4851) and GTR + I + G (AIC = 9579.5820; lnL = 4779.7910; freq A = 0.2163; freq C = 0.2034; freq G = 0.3165; freq T = 0.2638; R(a) = 0.7283; R(b) = 2.7572; R(c) = 1.3283; R(d) = 0.4052; R(e) = 5.6136; R(f) = 1; Pinva = 0.2046; Shape = 0.4580), respectively. Posterior probability values exceeding 50% are given for the appropriate clades. (**B**) Maximum Likehood Phylogeny inferred using a total of 299 one-to-one single copy orthologous by IQTREE v2 with 1000 bootstrap values under LG + F + R5 substitution model.

The phylogenetic relationships of 28 *Caenorhabditis* species and one outgroup species (*Prodontorhabditis wirthi*) inferred from the full length SSU rRNA and D2-D3 regions of LSU rRNA were congruent with previously provided phylogenetic trees, except for a few terminal nodes and the placement of *C. plicata*, which was as part of the *drosophilae* supergroup with low posterior-probability support^4,6^. *C. auriculariae* was placed close to *Caenorhabditis sonorae*, which was isolated from the rotten cactus *Carnegiea gigantea* in the USA^10^, and *Caenorhabditis monodelphis*, isolated from the galleries of the fungal-feeding beetle, *Cis nitidus* inside fruiting bodies of *Ganoderma applanatum*^3,4^. These three species formed an independent clade at the basal (outgroup) position of the other *Caenorhabditis* spp. (Fig. 2A). The GenBank accession numbers of the sequences compared are listed in Table S1.

### Voucher material

Ten male and 10 female *C. auriculariae* adults have been vouchered as permanent slides at the Forest Pathology Laboratory collection of the Forestry and Forest Products Research Institute with the material numbers *Caenorhabditis auriculariae* M01–10 and F01–10. The TAF-fixed materials and unmounted glycerol-processed materials have also been vouchered into the collection. Live cultures and a frozen stock of *C. auriculariae* has been deposited in Taisei Kikuchi’s Lab. (culture code NKZ352; Miyazaki University, Miyazaki, Japan), and further genomic and transcriptomic analyses will be conducted.

### Genome characteristics of *C. auriculariae*

For a deeper understanding of the phylogenetic status and biological features of *C. auriculariae*, we sequenced the genome of the species and conducted a genome comparison with other *Caenorhabditis* species. The hybrid assembly using Nanopore long reads and Illumina short reads (Table S2) resulted in a 109.5 Mb assembly composed of 491 scaffolds with high completeness values (89.8% BUSCO and 95.9/99.6% partial/complete CEGMA) (Table 2). A total of 16,279 protein-coding genes with a mean protein length of 435.34 and the largest of 8,188 amino acids were predicted on the genome assembly. This genome size is ~ 10% larger, but the predicted gene number is slightly smaller than those in *C. elegans*. The *C. auriculariae* genome contained of 20.8 Mb repetitive sequences that account for 19.04% of the genome, which is similar amount as *C. elegans* genome (18.45%) (Table 3). DNA repeat family was the most abundant (1.6%) followed by LINE family (0.91%) and LTR family (0.51%), though a large portion of *C. auriculariae* repeats (15.24%) were classified as “Unclassified”. Compared to *C. elegans,* more retroelements (LINEs and LTRs) were identified in *C. auriculariae,* which is consistent with the fact that the *C. auriculariae* gene models contained a higher number of transposon genes (Table 2) and RVT (reverse transcriptase) domains (see Pfam result below).

**Table 2:** Genome and gene model statistics for *C. auriculariae* and comparisons of other *Caenorhabditis* species.

Species	*C. auriculariae*	*C. monodelphis*	*C. angaria*	*C. japonica*	*C. elegans*	*C. inopinata*	*C. briggsae*
Taxonomic group	Basal group	Basal group	*Drosophilae* super group	*Japonica* group	*Elegans* group	*Elegans* group	*Elegans* group
Mating type	Gonochoristic	Gonochoristic	Gonochoristic	Gonochoristic	Hermaphroditic	Gonochoristic	Hermaphroditic
Version	v1.1	v1	WBPS15	WBPS15	WS276	WBPS15	WS271
Strain	NKZ352	JU1667	NA	DF5081	N2	NKZ35	AF16
Span (Mb)	109.5	115.1	105.9	156.5	100.2	122.9	108.3
Scaffolds (n)	433	6864	34,621	662	6	6	367
N50 (Mb)	0.95	0.05	0.07	0.86	17.49	20.59	17.49
Genes (n)	16,279	17,180	27,970	29,935	19,999	21,443	20,829
Num. transposon proteins	486	176	157	3578	114	3512	148
GC (%)	38.2	43.9	35.7	39.2	35.4	38.5	37.4
BUSCO %	89.8	89.8	77.9	92.4	98	96.6	97
CEGMA complete/partial %	95.97/99.60	93.95/ 97.58	79.44/95.16	80.65/ 96.37	98.39/100	98.79/100	99.19/100

**Table 3:** Repeat contents in *C. auriculariae* and *C. elegans*.

	*Caenorhabditis auriculariae*		*Caenorhabditis elegans*	
	num element	% in bp	num element	% in bp
SINEs	531	0.08	724	0.12
LINEs	2504	0.91	1041	0.49
LTR element	1770	0.51	1412	0.58
DNA element	5774	1.55	55,152	9.28
Rolling-circles	582	0.13	7289	1.88
Small RNA	174	0.02	551	0.05
Satellites	247	0.02	5326	1.44
Simple repeat	10,945	0.38	20,390	0.96
Low complexity	4000	0.16	5916	0.28
Unclassified	96,519	15.24	20,703	3.37
Total	19.04%		18.45%	

We then performed a phylogenomic analysis using 35 *Caenorhabditis* species whose draft genome sequences were available with *D. pachys* as an outgroup. A ML tree based on 97 single-copy genes showed a mostly consistent topology to the nuclear rRNA tree; species of *elegans* group and *japonica* group each formed a separated cluster, with species of *drosophila* supergroup located at more basal posidion of the tree. *C. auriculariae* was placed at the most basal position of *Caenorhabditis* genus with *C. monodelphis* (Fig. 2B). *C. parvicauda*, which has a morphological novelty, secondary loss of bursa, and is considered highly divergent^11^, shows a long branch in the tree but belongs to the inner clade.

**Figure 3:**
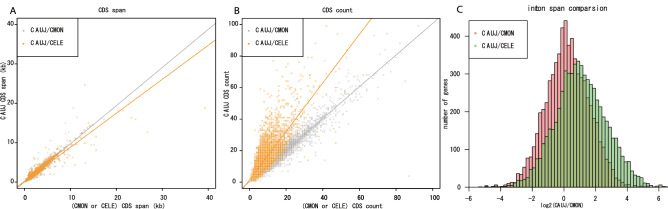
Comparison of gene structure in single-copy orthologues between *C. auriculariae*, *C. elegans* and *C. monodelphis*. Whereas total CDS length per gene is similar in the three species (**A**), *C. auriculariae* and *C. monodelphis* have larger CDS counts (**B**) and longer intron span per gene (**C**) than *C. elegans.* CAUJ; *C. auriculariae,* CMON; *C. monodelphis,* CELE; *C. elegans*.

The two basal species, *C. auriculariae* and *C. monodelphis,* showed similar genome statistics to each other. For instance, *C. auriculariae*/*C. monodelphis* total assembly size are 109.5/115.1 Mb and 16,279/17,180 in the predicted gene numbers (Table 2). The gene structures of *C. auriculariae* are also similar to those of *C. monodelphis,* in which genes are generally longer, contain more exons, and a longer span of introns than *C. elegans* genes (Fig. 3), which was suggested to reflect an ancestral status of *Caenorhabditis* genome structure^12^.

**Figure 4:**
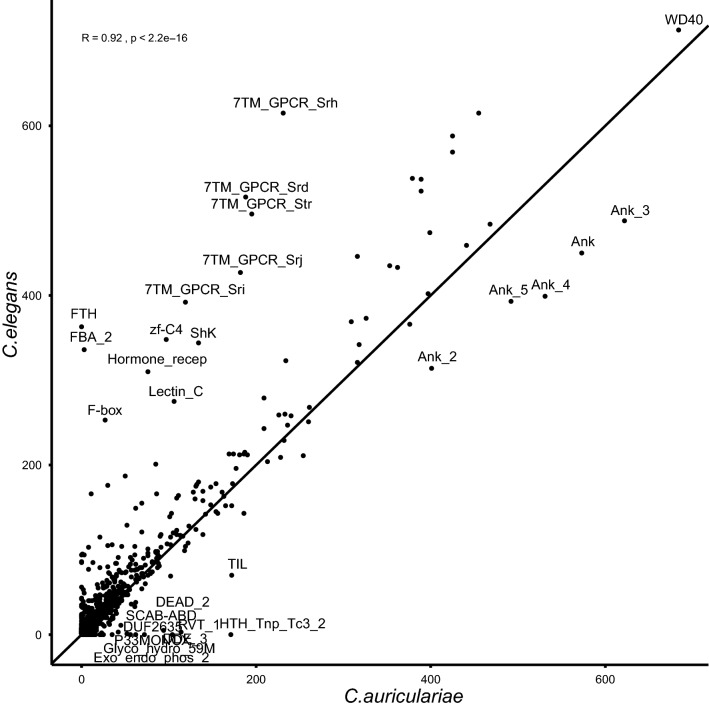
Pfam domain abundance in *C. auriculariae* and *C. elegans*. The x-axis represents abundance of Pfam domains in *C. elegans* and the y-axis represents abundance of the same domains in *C. auriculariae*. Linear regression is plotted alongside their respective equations and correlation coefficients. PFAM domains enriched were labelled with the domain names.

**Figure 5:**
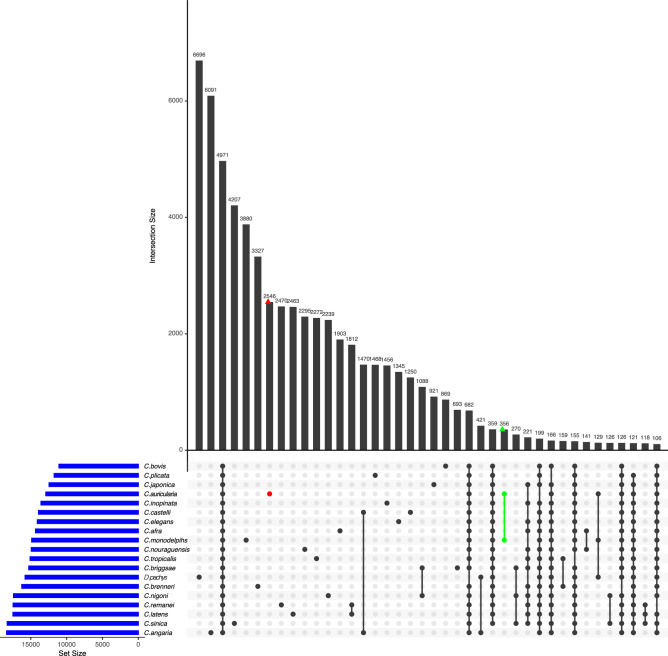
Orthologue comparison across the 18 *Caenorhabditis* and *D. pachys* genomes. UpSetR plot showing unique and overlapping protein ortholog clusters. The intersection matrix is sorted in descending order. Blue bars represent the orthogroup size for each genome and connected dots represent intersections of overlapping orthogroups while vertical bars show the size of each intersection. Orthogroups unique to *C. auriculariae* and *C. auriculariae-C. monodelphis* are shown in red and green, respectively.

Comparison of protein domain (Pfam) distribution patterns in the genomes revealed that *C. auriculariae*, compared to *C. elegans,* has higher numbers of Ank, LRR, HEAT, TIL, HTH_Tnp_Tc3_2, DDE_3, RVT_1, and DEAD protein domains. The numbers of protein domains related to receptors (GPCRs, Hormone_recep, and Recep_L_domain), WD40, Collagen, Ig_3, I-set, V-set, Pkinases, EGF, Zinc finger, Shk, C2-set_2, FTH, FBA_2, Lectin_C domains are smaller in *C. auriculariae* (Fig. 4). Gene family (orthologue) analysis assigned a total of 389,541 genes (90.8%) of 18 *Caenorhabditis* species and *D. pachys* into 31,748 orthogroups. Of 31,748 orthogroups, 4971 orthogroups were shared by all species. A high number of orthogroups (9737 orthogroups) are shared by *C. auriculariae* and *C. monodelphis* with 356 unique to the clade. However, the two species still exhibit high numbers of species-specific orthogroups: 2546 and 3880 orthogroups unique to *C. auriculariae* and *C. monodelphis*, respectively (Fig. 5). *C. auriculariae* specific-orthologous include genes encoding proteins with Ank (Ankyrin), TIL (Trypsin Inhibitor like cysteine rich domain), LEA_4 (Late embryogenesis abundant), GPCR (G protein-coupled receptors), Collagen, Pkinase (Protein kinase) and Apolipoprotein domains (Table S3), suggesting that genes of those functions are highly diverged in *C. auriculariae* and possibly reflecting its unique lifestyle though it has not been revealed yet.

**Figure 6:**
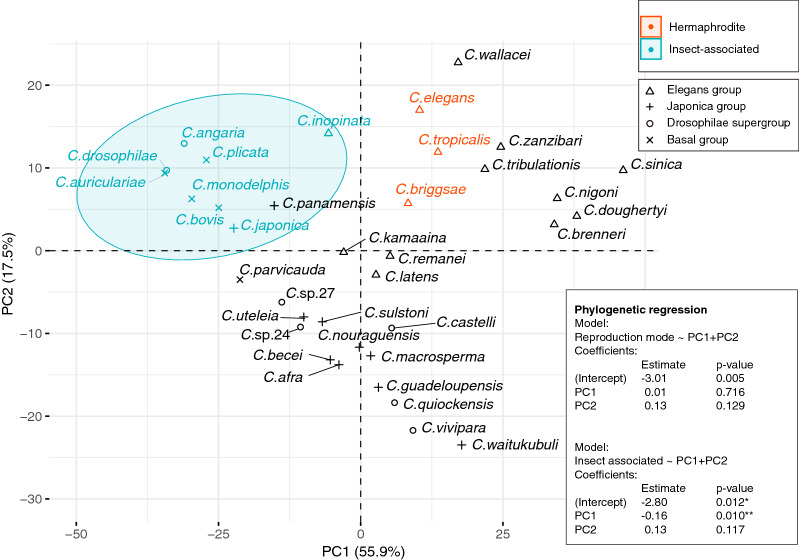
Principle component analysis (PCA) score plot of Carbohydrate active enzyme (CAZy) distribution. The first two axes explain 55.9% and 17.5% of total variance, respectively. Taxonomic groups and ecological traits (hermaphrodism and insect association) are indicated by point shapes and colours, respectively. The ellipse in the plot illustrates the 95% prediction region. The best models of phylogenetic logistic regression for the ecological traits with PCs were shown in box.

Carbohydrate-Active enzymes (CAZy) are involved in several biological processes, including feeding, energy metabolism, structural support, and signal transduction^13^. The repertories in the genome generally reflects its life style. We identified a total of 312 CAZy genes (5 auxiliary activities (AA), 32 carbohydrate-binding modules (CBM), 47 carbohydrate esterases (CE), 71 glycoside hydrolases (GH) and 157 glycosyltransferases (GT)) in *C. auriculariae*, which is a comparable number with other *Caenorhabditis* species (Table S4). We found many CAZy classes are common to the 35 *Caenorhabditis* species (e.g. two AA, seven CBM, four CE, 18 GH and 30 GT) though the number of genes in each class varies, but some are species or group specific (e.g. GH131 in *C. brenneri* and GH88 in *C. guadeloupensis*)*.* To reduce the complexity of the CAZy distribution patterns across 35 *Caenorhabditis* species, we conducted a principal component analysis (PCA). The first two principal components explained 73.4% of the overall variance (55.9% and 17.5%for principal component 1 and 2, respectively) (Fig. 6). The PCA plot (Fig. 6) clustered species largely by the taxonomic groups; species of *elegans*-group were mostly located upper right, most of *japonica-* and *drosophilae*-groups were placed lower middle, and the basal group was on the upper left. However, interestingly, this CAZy-based plot seems also highly correlated with particular lifestyles. For instance, *C. inopinata*, *C. japonica, C. drosophilae* and *C. bovis* were placed close to each other although they belong to *elegans*-group, *japonica*-group, the *drosophilae*-supergroup, and a separate basal clade, respectively. These four nematodes are well-known insect-associates as using insects as distributing vectors^14–16^. *C. angaria* and *C. castelli* are phylogenetically close to each other, but they were clearly separated by PC1 and PC2. Similarly, *C. angaria* has a tendency to ride weevils^17^ whereas there are no reports about an insect association for *C. castelli*. We have tested if there is a relationship between the trait (insect-association) and the CAZy distribution using the phylogenetic logistic regression with the PC values and found a significant correlation between PC1 and the trait (p < 0.01) (Fig. 6). It is also interesting to note that the hermaphroditic species (i.e., *C. elegans*, *C. briggsae* and *C. tropicalis*) were placed together in the PCA plot although those hermaphrodism were evolved independently in the *Caenorhabditis* evolutionary history although the regression test was not statistically significant (Fig. 6).

In the PCA plot, *C. auriculariae* was placed together with *C. monodelphis* on the top left, suggesting those basal species have similar lifestyles to each other and they possibly have tight associations with insects, which is consistent with the fact that they were isolated from beetles.

## Discussion

### Morphological comparison with other molecularly characterized *Caenorhabditis* spp

*Caenorhabditis auriculariae* was described in 1999 before the deep-level phylogeny of the genus or the relationship between morphological characteristics and phylogenetic status had been examined^9^. Later, Kiontke et al*.*^4^ examined nominal and undescribed *Caenorhabditis* spp. using multiple molecular loci and coded their typological characteristics in a phylogenetic analysis.

The genus *Caenorhabditis* was separated into two supergroups (*elegans* and *drosophila* supergroups) based on molecular phylogenetic analyses and male tail characteristics. In addition, there were several species that do not fall into those supergroups^4,6^ which tentatively regarded as basal group. Basal group species including *C. auriculariae* harbour some typical characteristics from both supergroups that are hypothesized to be the stem species pattern, namely: (1) oval and anteriorly opened bursa without serratae and terminal notch on the edge of the velum, (2) nine pairs of bursal rays in which p2 reaches to the edge of the velum, p2 and p3 are clearly separate, and p1 is directed dorsally, (3) precloacal lip is rounded, (4) spicule with a slightly ventrally bent blade and complex tip, and 5) parallel mating position^4^, although several species-specific apomorphies, e.g., the secondary loss of bursa in *C. parvicauda*^11^, has been reported. As are in other basal group species, several species-specific apomorphies (or clade, if there is a closely related cryptic species) are evident after comparing the morphological and molecular phylogenetic status of *C. auriculariae*. The typological characteristics of the *C. auriculariae* male tail are (1) wide, heart-shaped bursa with an anterior serrated-edge velum and no terminal notch, (2) nine pairs of bursal rays arranged as (p1d, p2)/P3, (p4 + p5d), p6, (p7m p8d), (ph, p9), where p2 does not reach to the edge of the velum and p2 and p3 are clearly separate, (3) precloacal lip forms a heart-shaped or bifid cap structure, (4) stout and evenly curved spicule with a complex spicule tip (possessing a dorsally oriented small projection at the distal tip), and (5) parallel mating position (not spiral).

*Caenorhabditis auriculariae* spicule morphology is similar to that of several *drosophilae* supergroup species (*C. drosophilae*, *C. angaria*, *C. castelli*, and *Caenorhabditis* sp. 2 and sp. 8) and *C. monodelphis*^4,6,10,17^. The bursal velum morphology is similar to all *elegans* supergroup species and several *drosophilae* supergroup species (*C. portoensis*, *C. virilis*, and *C. latens*)^4,6^. The arrangement of bursal rays is somewhat intermediate between the two supergroups. For example, the short p2 that does not reach the edge of the velum is similar to the *elegans* supergroup and *C. virilis*; dorsally directed p5d is shared with the *elegans* supergroup and *C. monodelphis*, clearly separate p2 and p3 are shared with all non-elegans group species, and dorsally directed p8d is shared with two *drosophilae* supergroup species (*C. portoensis* and *C. virilis*)^4,6^. Additionally, the parallel mating position is similar to all known *Caenorhabditis* except three *drosophilae* supergroup species (*C. angaria*, *C. castelli*, and *Caenorhabditis* sp. 8)^4,6^. The heart-shaped cap on the precloacal lip and the arrangement of the bursal rays (see above) are unique to *C. auriculariae*. In addition, the stomatal morphology of *C. auriculariae* is unique. Therefore, regardless of the unique characteristics, none of the nominal (and characterized) species exactly matched the typological characteristics of *C. auriculariae*. *C. auriculariae* is distinguished from all other phylogenetically characterized *Caenorhabditis* sp. based solely on typological characteristics.

The rRNA and genome-based phylogeny suggested the closeness of *C. auriculariae* with *C. sonorae*, and *C. monodelphis*, as these three species formed a well-supported independent clade at the basal position of the genus. They, however, can be clearly separated by their typological characteristics, as the bursal velum, ray characteristics, and precloacal lip structure differ from each other^4,10^. In addition, *C. auriculariae* has a quite unique stomatal morphology, with a long flap-like cuticular extension on the cheilostom and three bifid metastegostomatal teeth. Although the stomatal characteristics of *C. monodelphis* have not been described in detail, both species have a long and narrow stoma, and *C. sonorae* has a three triangular teeth, which is common in the genus, but *C. monodelphis* does not have glottoid apparatus^5,10^. The unique stomatal structure of *C. auriculariae* could be a species (or clade) specific apomorphy.

### Biological features and genome

*Caenorhabditis* nematodes have been isolated from many different environments and animals, such as rotting fruit^3,4,10^, rich soil and manure^18–21^, mushrooms^3,9^, insects^14,17,22^, soil and freshwater invertebrates^22,23^, and vertebrates potentially including humans^24–26^. Some vertebrate associations could be due to insect carriers associated with the “host” vertebrates. *C. monodelphis* and *C. auriculariae* were originally isolated from *G. applanatum* in Berlin, Germany and from *A. polytricha* in Kyoto, Japan, respectively, and are associated with fungal-feeding beetles^3,9^. In the present study, *C. auriculariae* was isolated from a fungal beetle, *Platydema* sp. Although the detailed carrier association, e.g., the beetle species is primary carrier of *C. auriculariae*, beetle body organ harbouring the nematode, and number and association rate of nematode in individual beetles, was not clarified in this study, at least the ability of insect association (phoresy) was confirmed for *C. auriculariae*. Because of the limited data, we cannot conclude that the fungal (mushroom) associations of these species are related to clade-specific habitat preferences or carrier insects. However, the present results will be useful to further isolate strains of those species. Diplogastrids nematodes, *Pristionchus* spp. were considered as the soil-inhabiting free-living nematodes for long time, and its close insect association has been confirmed recently^27,28^. The close rotten fruits-association of *Caenorhabditis* spp. has not been recognized for a long time^4^. After findings of these associations, the number of new species isolation increased dramatically^3,4,29^. Similarly, this study and recent reports on insect-associated *Caenorhabditis* spp. should enhance new species identification of the genus by surveys of nematodes around insects.

The genome comparison revealed the presence of highly diverged or unique genes encoding GPCRs in *C. auriculariae*. GPCRs work as primary receptors to detect a wide variety of environmental signals and are therefore highly diverged in organisms or even among individuals^30,31^. The unique repertoires of *C. auriculariae* GPCRs probably reflect the need to detect environmental signals specific for its lifestyle, such as mushroom and insect associations. The genome comparison also found diverged LEA proteins in *C. auriculariae*. LEA proteins were initially discovered accumulating late in embryogenesis of cotton seeds and later shown to have a role to protect proteins against aggregation due to desiccation or osmotic stresses in some plants, bacteria and invertebrates^32,33^. Further functional investigation is necessary, but this may reflect its life-cycle in which the nematode encounters relatively dry condition compared to *C. elegans*.

CAZy distribution-based PCs separated insect associated species from non- or less- associates regardless of their phylogenetic relationships. Furthermore, this method roughly separated hermaphroditic species from gonochoristic even when two closely related sister species have contrastive reproduction modes. Therefore, this method can be of particular usefulness to speculate on the lifestyle of newly isolated species with non-detailed ecological information. For example, based on the fact that *C. pamanensis* was placed in the insect-associate ellipse (Fig. 6), we could speculate that the worm has a lifestyle with a tight insect-association, although no such records were reported (Table 1). Indeed, there are several rare *Caenorhabditis* species with unclear ecological status, such as *C. yunguensis* (Table 1).

This study provided a high-quality genome reference for *C. auriculariae.* A genome of *C. monodelphis* was recently published as an outgroup reference for *Caenorhabditis*^12^. *C. auriculariae* is also phylogenetically placed at the basal position of the genus and shared several genome features with *C. monodelphis*. However, the distance of the two species is substantially long, and each genome contained a number of species-specific genes. Therefore, *C*. *auriculariae*, together with *C. monodelphis,* provides a powerful resource to perform deep evolutionary studies in the genus *Caenorhabditis*.

## Methods

### Nematode materials

Potential carrier insects of nematodes were collected in the field in Nagoya, Aichi, Japan on 17 June 2015. The samples were collected under an official permit from the Nagoya City local governmental office. Several species of coleopteran insects (beetles) were collected, brought back to the laboratory, morphologically identified, and dissected to examine their association with nematodes. The dissected insect bodies were placed in 2.0% water agar to allow propagation of phoretic microbe-feeding species and examined occasionally. No endangered or protected species were collected in the present study.

A *Caenorhabditis* sp. was isolated from the dissected body of *Platydema* sp. (Coleoptera: Tenebrionidae); the nematode was not confirmed during the dissection but propagated on the dissected body of its carrier beetle. The nematode was observed under a light microscope (Eclipse 80i: Nikon, Tokyo, Japan) to determine its feeding habits. It was then transferred to nematode growth medium (NGM) and kept as a laboratory strain with culture code NKZ352.

### Morphological observations and micrographs

Live and TAF-fixed *C. auriculariae* material from 2-week-old cultures was observed under a light microscope using the methodologies defined by Kanzaki^34^. The nematode were identified to species based on typological characteristics when compared with the original description^9^. Thereafter, the TAF-fixed material was processed into glycerin according to a modified Seinhorst’s method^35^ and deposited as morphological vouchers.

Several morphological characteristics that were not provided in the original description, e.g., detailed stomatal structure, were drawn using a drawing tube, and other general characteristics were photo-documented using a digital camera system (DS-Ri1, Nikon) connected to a microscope.

### Scanning electron microscope (SEM) observation

For SEM observation, adult nematodes were treated with the pre-fixation solution (2% paraformaldehyde, 2.5% glutaraldehyde, 0.1 M Cacodylate, pH 7.4) for 2 h at 4 °C followed by incubation in the fixation solution (1% OsO4, 0.1 M Cacodylate, pH 7.4) for 1 h at 4 °C. Samples were then dehydrated in ethanol (50% to 100%, gradually). They were substituted by isoamyl acetate and were dried by using a freeze-drying device (Eiko ID-2). Dried nematodes were coated with Platinum by using ION SPUTTER (HITACHI E-1045) and were observed by using SEM (Hitachi S-4800) operating at 20 kV.

### Molecular profiles and preliminary phylogenetic analyses

Prior to genome wide phylogenetic analysis of selected species, the phylogenetic status of *C. auriculariae* within the genus was analysed based on the ribosomal RNA genes. Nematode lysate material was prepared for use as a polymerase chain reaction (PCR) template according to the protocol developed by Kikuchi et al*.*^36^ and Tanaka et al.^37^. The molecular sequences of small subunit ribosomal RNA (SSU rRNA) and D2-D3 regions of large subunit ribosomal RNA (LSU rRNA) were sequenced with the PCR direct sequencing methods developed by Ye et al*.*^38^ and Kanzaki and Futai^39^.

A Bayesian molecular phylogenetic analysis was conducted based on SSU and D2-D3 LSU as previously described^40^. The sequences were aligned using MAFFT^41^ and the base substitution model was determined using Modeltest ver. 3.7^42^ under the Akaike information criterion model selection criterion. Then, a Bayesian analysis was performed to infer the tree topology of each gene using MrBayes 3.2^43^; four chains were run for 4 × 10^6^ generations. Markov chains were sampled at intervals of 100 generations^44^. Two independent runs were performed, and the remaining topologies were used to generate a 50% majority-rule consensus tree after confirming convergence of runs and discarding the first 2 × 10^6^ generations as burn-in.

### DNA/RNA isolation and sequencing

For whole genome analyses, nematodes were propagated on NGM plates implemented with *E. coli* Op50 strain. After 2 weeks of incubation at 20 °C, nematodes were collected from the plate, washed five times with M9 buffer and the genomic DNA was extracted using Genomic-tip (Qiagen) following the manufacturer’s protocol. Paired-end and Mate-pair sequencing libraries were prepared using the Nextera DNA Sample Prep kit (Illumina) and TruSeq DNA Library Preparation kit, respectively, according to the manufacturer’s instructions and sequenced using Illumina MiSeq sequencer with the v3 kit (301 cycles × 2 or 76 cycles × 2) (Illumina) (Supplementary Table S2).

Two μg of genomic DNA was used to prepare Nanopore sequencing library using the Ligation Sequencing Kit SQK-LSK109 (Oxford Nanopore Technologies) according to the manufacturer’s protocol. The library was sequenced with a single 24 h run with FLO-MIN106 R9 MinION flowcell (Oxford Nanopore Technologies). Base calling for R9 runs was performed with Guppy v.3.1.5 using the ‘dna_r9.4.1_450bps_fast’ model and obtained 771,594 reads (~ 3 Gb) (Supplementary Table S2).

For mRNA-seq analysis, RNA was extracted from fresh mixed-stage nematodes using TRI reagent according to the manufacturer’s instructions. Total RNA samples were qualified using Bioanalyzer 2100 (Agilent Technology, Inc.) and only samples with an RNA integrity value (RIN) greater than 8.0 were used for library constructions. One hundred ng of total RNA was used to produce an Illumina sequencing library using the TruSeq RNA-seq Sample Prep kit according to the manufacturer's recommended protocols (Illumina). The RNA libraries were sequenced using Illumina MiSeq sequencer with the v3 kit (301 cycles × 2) (Illumina) (Supplementary Table S2).

### Genome assembly

Three de novo assemblers were used to generate initial assemblies. The Nanopore reads (~ 771 K reads, N50 = 4.7 kb) were assembled with Flye (v.2.7.1)^45^ in raw nanopore mode using -g 100 m or Canu^46^ using genomeSize = 100 m, both followed by base correction by Illumina DNA reads using Pilon (v.1.22)^47^. Spades (v.3.7.1)^48^ was separately used to generate a hybrid assembly of Nanopore, Illumina pair-end and mate-pair reads (Supplementary Table S1) with the default options after trimming of Illumina reads for after trimming for low quality and adaptor contamination using Trimmomatic (v.0.32)^49,50^. The three assemblies were merged using MetaAssembler (v.1.5)^51^ with the Flye assembly as a reference. Haplomerger2 (20151106)^52^ was run on the merged assembly to remove remaining haplotypic sequences. Further base corrections were performed by ICORN2^53^ using ~ 5G base of the Illumina pair-end reads. Contigs derived from bacteria or other organisms contaminations were identified and removed from the assembly using Blobtools^54^ and BlastN search against NCBI bacterial nt database. CEGMA v2^55^ were used to assess the completeness of the assemblies.

### Gene prediction

RNA-seq read pairs were aligned to the *C. auriculariae* assembly using Hisat2 v2.1.0^56^ with default parameters and used to generate intron hints using bam2hints script in Augustus v3.3.2^57^. Protein-coding genes on the assembly were predicted using BRAKER2^58^ with the intron hints and protein homology hints from ~ 78,000 proteins of 9 nematode species (*Brugia malayi, Bursaphelenchus xylophilus, Caenorhabditis elegans, C. briggsae, Necator americanus, Pristionchus pacificus, Strongyloides ratti, Trichinella spiralis,* and *Trichuris muris*).Protein domain annotations were performed on the gene models using Pfam search (ver. 28.0)^59^ with HMMER v3.1b2^60^ with e-value cutoff (1e−5).

### Carbohydrate-active enzyme analysis

Carbohydrate-active enzyme (CAZy) were detected using CAZy database^61^ and HMMER v3.1b2 under e-value cutoff (1e−5). Possible contaminations of bacteria or fungi were removed from the detected CAZy genes using BlastP search results against NCBI nr database. CAZy genes of each species were then counted for auxiliary activities, carbohydrate-binding modules, carbohydrate esterases, glycoside hydrolases, polysaccharide lyases and glycosyltransferases, separately.

Principal component analysis was performed for CAZy distribution of 35 *Caenorhabditis* species using the prcomp function and the results were visualised by Factoextra package^62^ both implemented in R (https://www.r-project.org/). Phylogenetic logistic regressions for ecological traits (reproduction modes or insect-associations) were performed with the Phylolm R package^63^ using the principal component values (PC1 to PC4) as explanatory variables and the tree shown in Fig. 2B as phylogenetic information under the logistic_IG10 method and the best models were selected by Akaike's entropy-based Information Criterion (AIC).

### Orthologous relationship of *C. auriculariae* with other *Caenorhabditis* species and constructing phylogenetic tree

Orthologous analysis of *C. auriculariae* with 17 selected *Caenorhabditis* species and *Diploscapter pachys* as an outgroup was performed using OrthoFinder v2.3.11^64^ with default parameters using the longest isoform set of each species. Orthologous distribution among species was visualised using the UpSetR R package^65^.

For a genome-wide phylogenetic analysis, amino acid sequences of 96 single-copy orthologous in 37 species were aligned using MAFFT v7.221^41^ with auto options. Poorly aligned regions were removed using Gblocks v0.91b^66^ with the parameters (-t = p, -b4 = 10, -b5 = n, -b6 = y, -s = y, -p = y, -e = -gb). The alignments were concatenated and used to generate a maximum-likehood tree using RAxML v8.0.26^67^. For the RAxML analysis, alignments were partitioned by gene with the PROTGAMMAAUTO model (the best-fitting model for each gene) used for all partitions. The topological robustness was assessed with 100 replicates of fast bootstrapping. Resulting phylogenetic tree was visualized in FigTree v1.4.4^68^.

## Supplementary Information

Supplementary Information 1.

Supplementary Information 2.

## Data Availability

The raw sequencing data have been deposited to the DNA Data Bank of Japan Sequence Read Archive under the BioProject PRJDB10634. The *C. auriculariae* assembly was deposited in the DDBJ/EMBL/GenBank under Project PRJEB40642 (https://www.ebi.ac.uk/ena/browser/view/ PRJEB40642).
