# Supplementary material for: Additional description and genome analyses of Caenorhabditis auriculariae representing the basal lineage of genus Caenorhabditis
Source: Sci Rep. 2021 Mar 24;11:6720. doi: 10.1038/s41598-021-85967-z (PMC7991662; doi:10.1038/s41598-021-85967-z)
Supplement: Supplementary file 2 — Supplementary Information 2. [file 41598_2021_85967_MOESM2_ESM.docx]

**Supplementary Text for**

**Additional description and genome analyses of *Caenorhabditis auriculariae* representing the basal lineage of genus *Caenorhabditis***

**Mehmet Dayi, Natsumi Kanzaki, Simo Sun, Tatsuya Ide, Ryusei Tanaka, Hayato Masuya, Kimiko Okabe, Hisashi Kajimura & Taisei Kikuchi**

Remarks on morphologically described species

Ten of the 37 nominal *Caenorhabditis* species have been described based on morphological characters, including *C. anthobia* (Schneider), *C. avicola* Schmidt & Kuntz, *C. clavopapillata* (Kreis & Faust), *C. craspedocercus* (Völk), *C. formosana* (Yokoo & Okabe), *C. fruticicolae* (Shinohara), *C. genitalis* (Scheiber), *C. oncomelaniae* (Yokoo & Okabe), *C. perrieri* (Maupas), and *C. chinkari* Mondal & Manna. In addition, no live cultures or molecular profiles are available.

The nominal and characterized species are listed in Supplemental Table S2 and their typological characters are summarized in Table S3 according to the species list and typological characters provided by Kionetke et al. ^1^ and Sudhaus and Kiontke ^2^. However, the lengths of the bursal rays (regardless of whether they reached the edge of the velum) and the edge of the bursal velum may not have been drawn or described precisely in many descriptions, particularly those before the 1970s. For example, none of the previously described species has a terminal notch on the bursal velum. Therefore, relatively clearly described or drawn characters, e.g., distance between p2 and p3 and overall spicule shape, are emphasized as depicted characters. In some cases, several micrographs were provided for the description, but may differ from the drawings or descriptions. In these cases, we relied on the micrographs to estimate the typological characters.

*C. anthobia* was initially described from water in *Cyrtandra glabra* (Gesneriaceae) inflorescences from South Africa ^3^. In the original description, drawings for pharyngeal region, female tail and male tail are provided, and former two do not give the group-level taxonomic information. In the description text and male tail drawing, only eight pairs of rays are reported. According to the general pattern of the arrangement of bursal rays, there are two possibilities, i.e., either p3 of *elegans* supergroup was missed, or the number of posterior rays were incorrectly counted. The p3 is relatively long and obvious in the genus, and thus, we consider later possibility. Then, based on the original drawing of male tail characters, the species probably belongs to the *drosophilae* supergroup and is probably closely related to the *C. portoensis*, *C. virilis*, and *C. guadeloupensis* clade, because the bursal velum is closed, i.e., anterior edge of the velum is pointed anteriorly, p2 ray does not reach the edge, p2 and p3 rays are clearly separate, and the spicule is long and slender with a slightly bent blade and complex tip; the male tail is drawn only from the lateral view ^1,3,4^.

*C. avicola* was initially described from the small intestine of the plumbeous water redstart, *Rhyacornis fuliginosus affinis* Vigors (Muscicapidae) from Taiwan ^5^. Based on the original drawings, the species belongs to the *elegans* supergroup because the bursal velum is closed, i.e., anterior edge of the velum is pointed anteriorly in lateral and ventral views, p2 bursal ray is clearly short and does not reach the edge, p2 and p3 are near each other, and the spicule is long and slender with an evenly curved blade and simple, pointed tip ^1,4,5^. Furthermore, the hook-like precloacal structure is clearly drawn in the lateral view of the male tail ^5^, which fits a character in the *elegans* supergroup.

*C. clavopapillata* was first described from feces-soiled hair in the perianal region of a dog from the USA ^6^. The detailed morphology of the bursal velum, e.g., open/closed and presence/absence of serratae and terminal notch, was not described. However, based on the original drawings, the species probably belongs to the *elegans* supergroup because the p2 and p3 bursal rays are close to each other and the spicule is long and slender with an evenly curved blade and a simple, pointed tip ^1,4,6^. Also, its relatively short p2 ray fits a character describing the *elegans* supergroup. The long p5 reaching the edge of the velum in the original description suggests it may be directed dorsally.

*C. craspedocercus* was described from an earthworm species, *Eisenia rosea* (Savigny) (Lumbricidae) from Germany ^7^. The detailed male tail characters were not described or drawn, but the closeness of the p2 and p3 rays, i.e., 3 + 3 + 3 arrangement described in the text, suggests that the species belongs to the *elegans* supergroup ^1,4,7^.

*C. formosana* was first described from the freshwater snail *Oncomelania hupensis formosana* Gredler (Rissooidea) in Taiwan ^8^. The morphological characters are difficult to interpret from the original description. According to the original drawings, the p1 and p2 rays are clearly separate and the bursal velum is open with a smooth edge, which are characters similar to several *drosophilae* supergroup species and outgroup clades, e.g., *C. drosophilae* and *Caenorhabditis* spp. 1 and 2 ^1,8^. However, the bursal velum was closed based on the micrographs, i.e., anterior edge of velum is pointed anteriorly in the lateral view, although the pictures are poorly resolved and it was difficult to confirm the arrangement of the rays and the edge of the velum. In contrast, the spicule is long and slender with an evenly curved blade and pointed tip ^8^; thus, the species may belong to the *elegans* supergroup.

*C. fruticicolae* was initially isolated from the intestine of the terrestrial snail, *Fruticicola* (*Acusta*) *sieboldiana* (Pfeiffer) (Helicoidea) from Japan ^9^. The species has 10 pairs of bursal rays in a 2 + 1 + 3 + 4 arrangement, but the one of posterior four pairs could be misinterpreted as a tail spike or a phasmid. According to the original description, drawings and micrographs, this species seemingly to belongs the *elegans* supergroups. The wide, heart-shaped bursal velum and short p2 ray are often found in the *elegans* supergroup and some *drosophilae* supergroup species ^9^. Spicule and gubernaculum morphologies are not clearly drawn or micrographed, and are not clearly consistent to each other. Thus, according to the micrograph, the spicule is relatively slender and straight, which is similar to elegans group species ^9^. The distances among the p2, p3 and p4 rays were not clearly described and differed between the lateral and ventral view drawings, i.e., rays are close to each other in ventral view and farther apart in lateral view ^9^. However, p3 is not clearly clustered with p4-6 in both drawings ^9^, suggesting the pattern is close to *elegans* supergroup. In contrast, the direction of the bursal rays is described as “rays 1, 3, 5, 6, 8, 9, and 10 reach progressively closer to the outer edge, and 2, 4, and 7 are close to the inner edge” ^9^. The inner (ventral) and outer (dorsal) surfaces could be a typo in the publication process. If true, dorsally oriented p4 and p7 are common to several *drosophilae* supergroup species. However, because of the uncertainty of the typological characters, the current intrageneric grouping is considered speculative and is not confirmed here.

*C. genitalis* was originally described from the outer genitals of a bedfast woman from Hungary ^10^. The species description was very old and the species (group/super group)-specific characters are not clearly described. According to the drawings, overall spicule shape was long and slender and the p2 and p3 rays were close to each other, i.e., bursal rays arranged as 3 + 6 and p1–3 rays are gathered around the cloacal opening, suggesting the closeness of p2 and p3. The bursal velum was oval-diamond shaped, which is similar to some *drosophilae* supergroup species. However, most of the typological characters are uncertain for this species; thus, the current intrageneric grouping is speculative. This species is hypothesized to be carried by insects inhabiting human-related environments based on the original source of isolation.

*C. oncomelaniae* was first isolated from a laboratory culture of the freshwater snail *Oncomelania hupensis nosophora* Gredler (Rissooidea) maintained at Kurume University, School of Medicine, Kurume, Japan, but the origin of the laboratory population was not specified ^8^. Therefore, the original locality for the nematode is assumed to be somewhere on Kyushu Island, Japan. Based on the original drawings and micrographs, the species belongs to the *elegans* supergroup, because the p2 ray does not reach the edge of the velum, p2 and p3 are close to each other, the bursal velum has a serrated edge, and the spicule is long and slender with an evenly curved blade and a simple, pointed tip ^1,8^.

*C. perrieri* was first described from soil at the bottom of a pile of manure in Algeria ^11^. The male tail of the species was described as having a closed bursa with a wavy (serrated?) velum edge and nine rays, p2 did not reach the edge of the velum, p2 and p3 were close to each other, and there was a long and slender spicule with an evenly curved blade and a simple, pointed tip. Furthermore, a pointed precloacal hook was clearly drawn and described. These features clearly fit the *elegans* supergroup ^1^.

*C. chinkari* was first isolated from feces of a captive chinkara, *Gazella gazella bennettii* (Sykes) (Bovidae), in West Bengal, India ^12^. According to the original description, the species has a hook-shaped precloacal lip, bursal velum with a smooth edge, nine pairs of bursal rays, where p1–3 are oriented dorsally and p2 and p3 clearly apart, and a long and slender spicule with an evenly curved blade and pointed tip ^12^. Furthermore, a pointed precloacal hook is described. The bursal velum was closed without a clear terminal notch. The anterior end of the bursal velum may be narrowly opened with a wavy edge, judging from the micrograph ^12^. Therefore, this species probably belongs to the *drosophilae* supergroup ^1,4^.

Re-isolating previously described species and conducting morphological re-characterizations and molecular characterizations are necessary to construct a more comprehensive model system.

**References:**

1 Kiontke, K. C. *et al.* A phylogeny and molecular barcodes for Caenorhabditis, with numerous new species from rotting fruits. *BMC evolutionary biology* **11**, 339 (2011).

2 Kiontke, K. & Sudhaus, W. Ecology of Caenorhabditis species. *WormBook* **9**, 1-14 (2006).

3 Schneider, W. *Freilebende Nematoden der Deutschen Limnologischen Sundaexpedition nach Sumatra, Java und Bali*. (1937).

4 Felix, M. A., Braendle, C. & Cutter, A. D. A streamlined system for species diagnosis in Caenorhabditis (Nematoda: Rhabditidae) with name designations for 15 distinct biological species. *PLoS One* **9**, e94723, doi:10.1371/journal.pone.0094723 (2014).

5 Schmidt, G. & Kuntz, R. Caenorhabditis avicola sp. n.(Rhabditidae) found in a bird from Taiwan. *Proceedings of the Helminthological Society of Washington* **39**, 189-191 (1972).

6 Kreis, H. A. & Faust, E. C. Two new species of Rhabditis (Rhabditis macrocerca and R. clavopapillata) associated with dogs and monkeys in experimental Strongyloides studies. *Transactions of the American Microscopical Society* **52**, 162-172 (1933).

7 Volk, J. *Die Nematoden der Regenwurmer und aasbesuchenden Kafer*. (G. Fischer, 1951).

8 Yokoo, T. & Okabe, K. Two new species of genus Rhabditis (Nematoda: Rhabditidae) found in the intermediate host of Schistosoma japonica, Oncomelania hupensis nosophora and Oncomelania hupensis formosana. *Agricultural Bulletin of Saga* **43**, 69-78 (1968).

9 Shinohara, T. Studies on Rhabditis (Nematoda, Rhabditidae). I. Rhabditis spp. obtained from human faeces. II. Rhabditis spp. found in the alimentary organs of Fruticicola (Acusta) sieboldiana Pfeiffer and Limax (Limacus) flavus Linné. *Journal of the Kurume Medical Association* **23**, 2777-2819 (1960).

10 Scheiber, S. Ein Fall von mikroskopisch kleinen Rundwürmern—Rhabditis genitalis—im Urin einer Kranken. *Archiv für Pathologische Anatomie und Physiologie und für klinische Medicin* **82**, 161-175 (1880).

11 MAUPAS, E. Modes et formes de reproduction des nematodes. Arch. Zool. Expt. e. *Gen* **8**, 578-582 (1900).

12 Mondal, S. & Manna, B. in *Proceedings of the Zoological Society.* 52-57 (Springer).


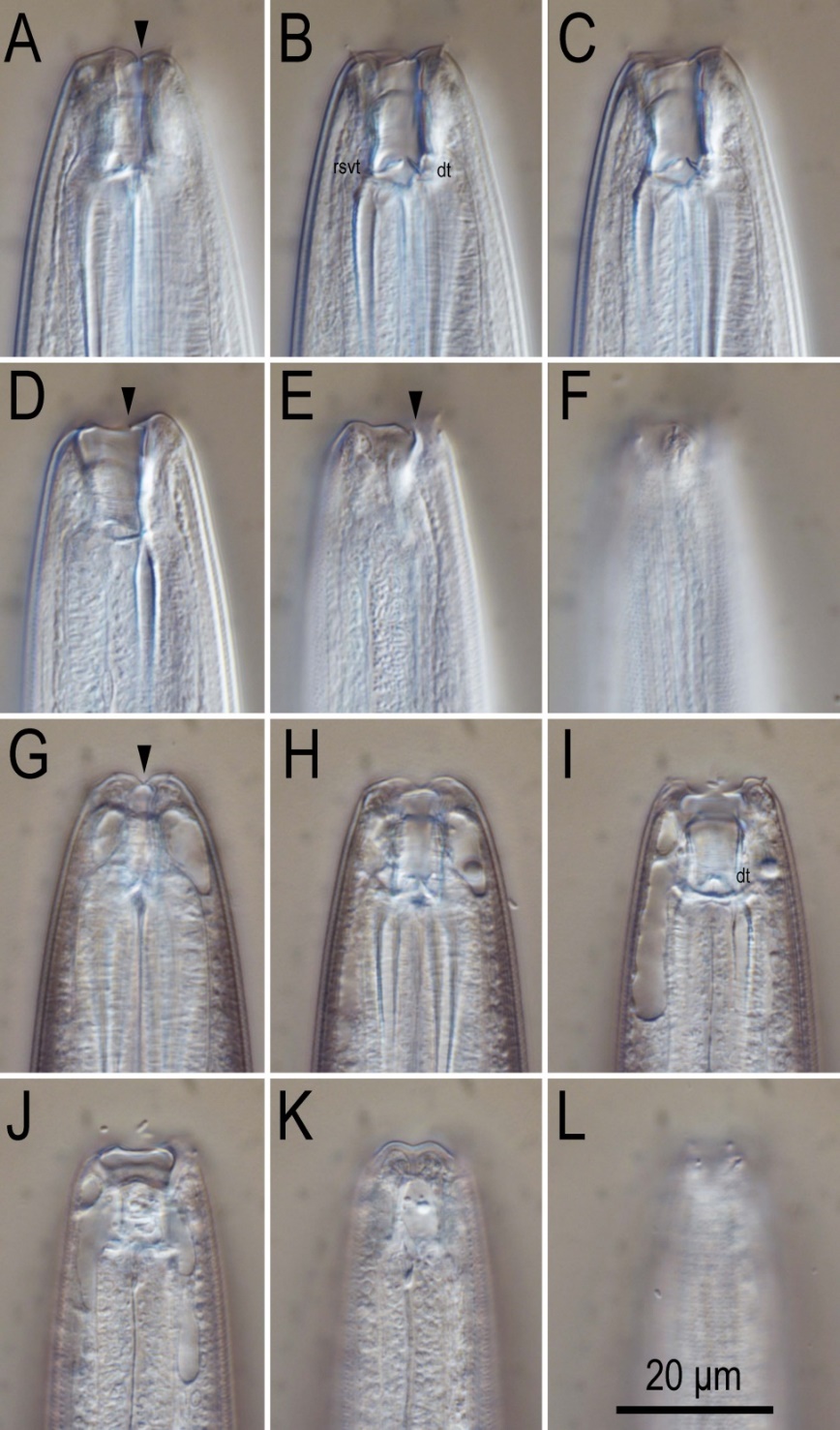


**Figure S1.** Stomatal morphology of *Caenorhabditis auriculariae*.

A-F: Left lateral view of the stomatal region in six different focal planes; G-L: Ventral view of the stomatal region in six different focal planes. Right subventral tooth (rsvt) is suggested in B, and dorsal tooth (dt) is suggested in B and I..


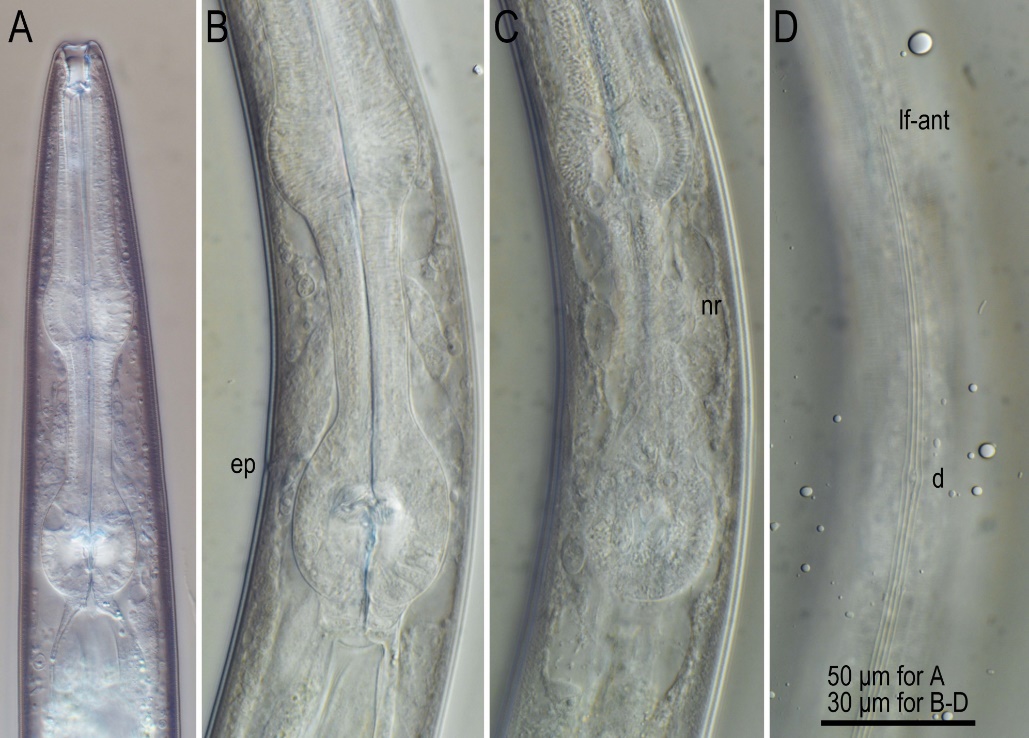


**Figure S2.** Anterior body of *Caenorhabditis auriculariae*.

A: Stoma and whole pharynx in right lateral view; B–D: median bulb (metacorpus) to cardia in different focal planes. The labels “ep”, “nr”, “lf-ant”, and “d” indicate the excretory pore, nerve ring, anterior end (starting point) of the lateral field, and deirid, respectively.


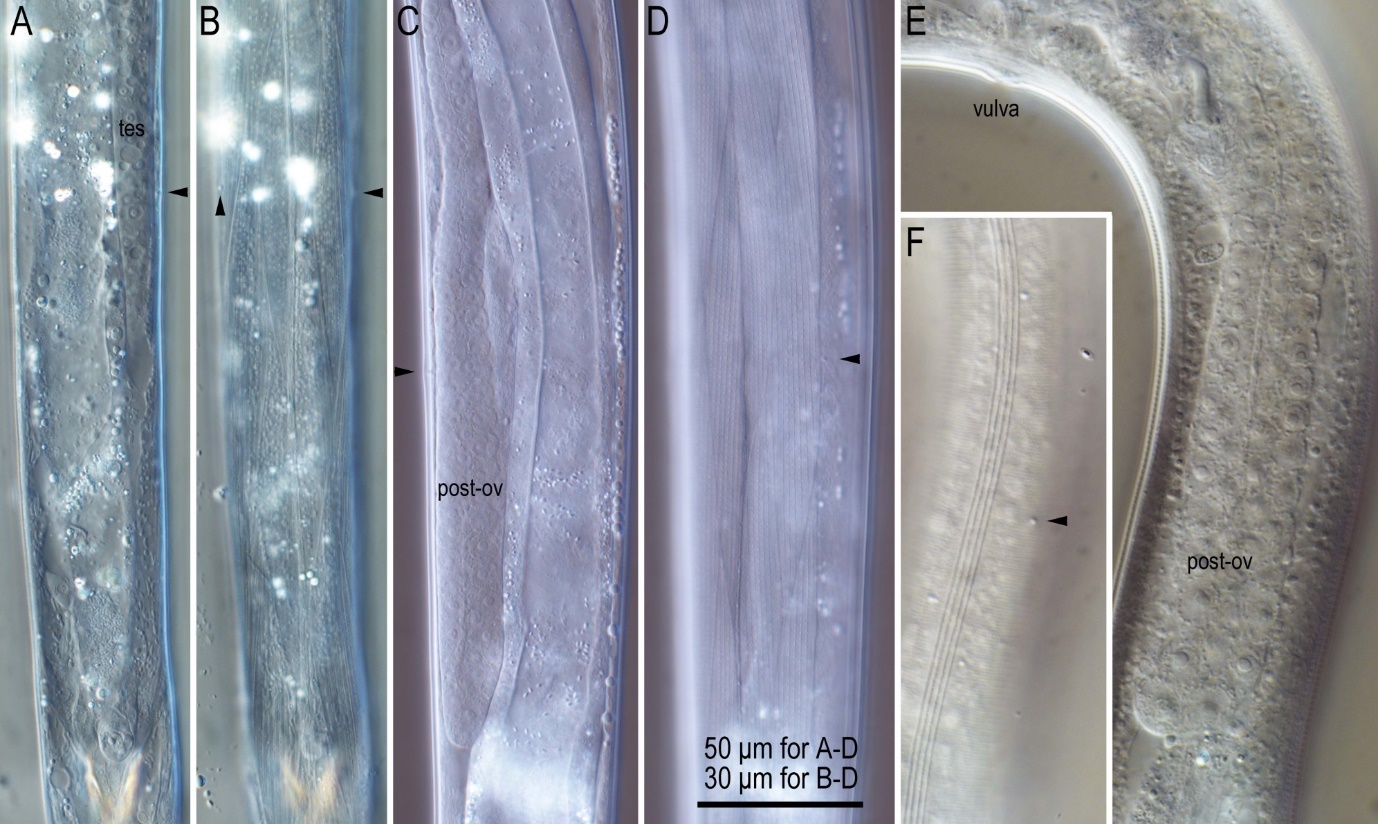


**Figure S3.** Postdeirid of *Caenorhabditis auriculariae*.

A, B: Ventral view of the male posterior region in different focal planes. C, D: Ventral view of the female posterior ovary region in different focal planes. E, F: Left lateral view of the female posterior ovarian region in different focal planes. Arrowhead, “tes”, “post-ov”, and “vulva” indicate Postdeirid, testis, posterior ovary, and vulval opening, respectively.


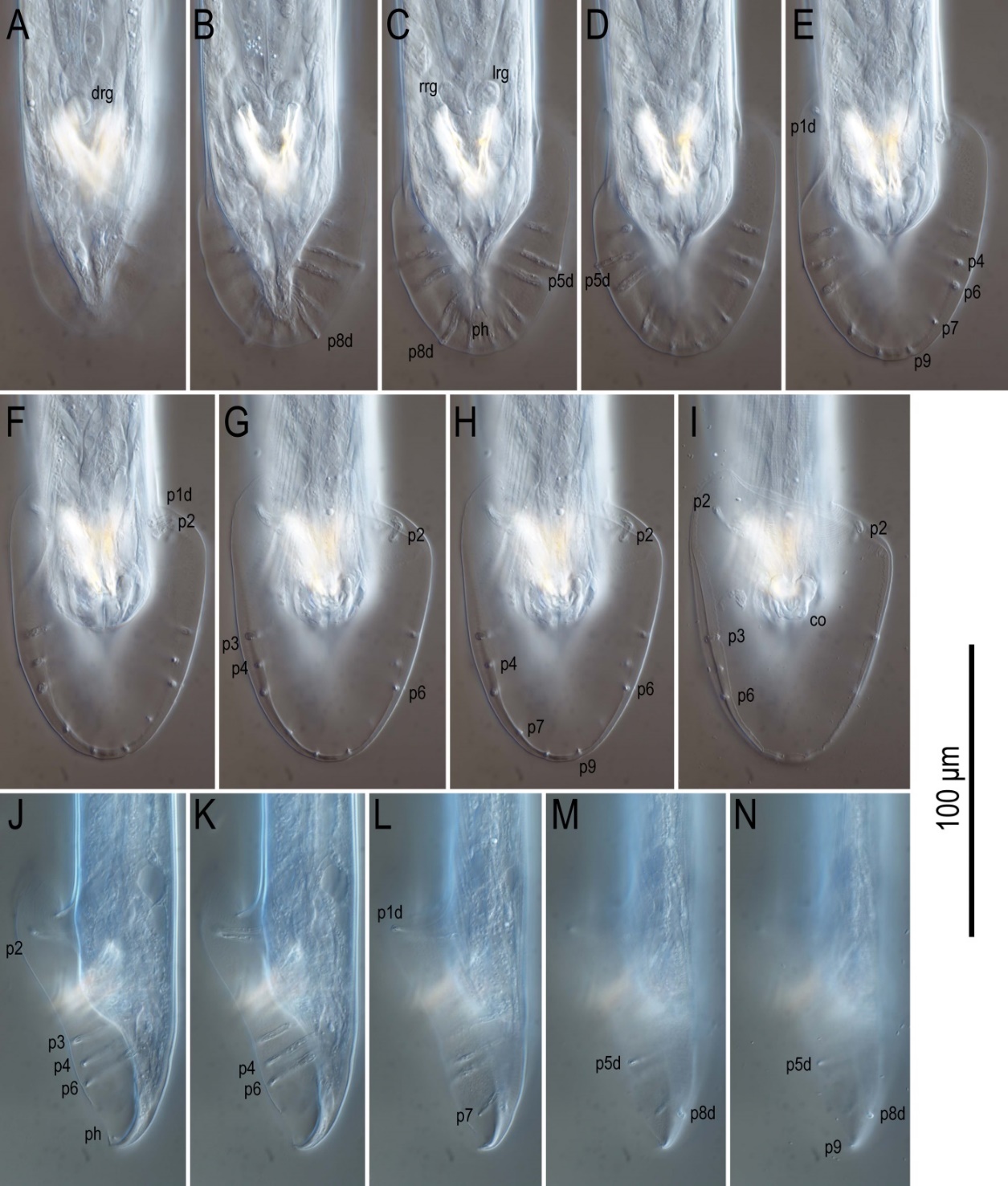


**Figure S4.** Male tail region of *Caenorhabditis auriculariae*.

A–I: Ventral view in different focal planes focusing through the dorsal to the ventral side; J–N: left lateral view in different focal planes focusing through the middle part to the left lateral surface. The labels “drg”, “rrg”, “lrg”, “ph”, and “p+number” indicate the dorsal rectal gland, right rectal gland, left rectal gland, phasmid, and bursal rays (genital papillae), respectively. Dorsally directed rays (papillae) are indicated with “d” after the numbers.


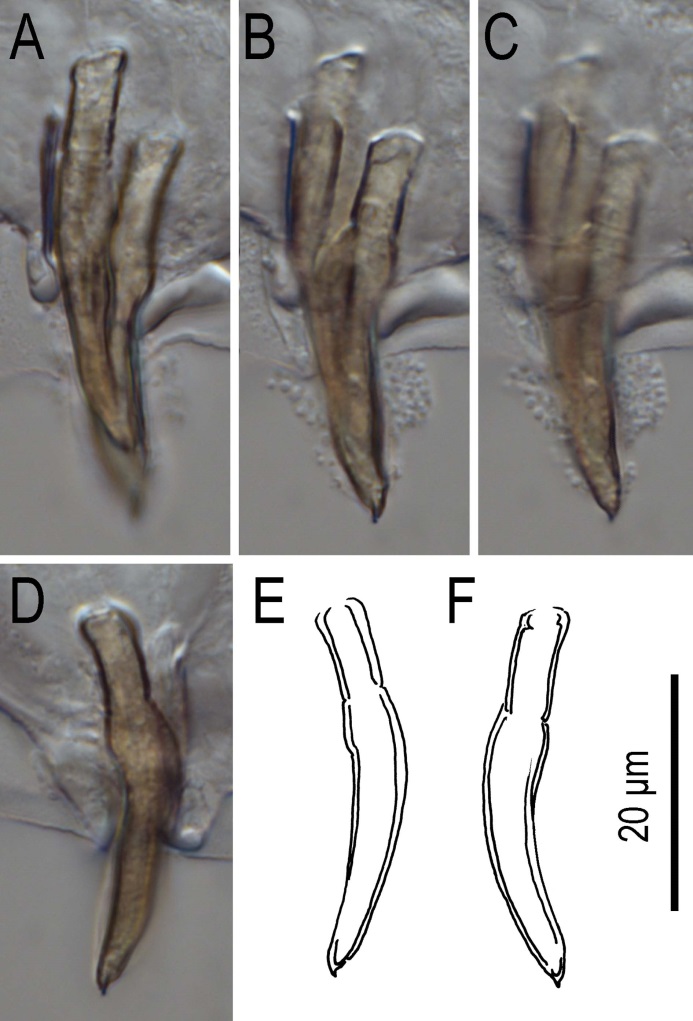


**Figure S5.** Close-up of male spicule of *Caenorhabditis auriculariae*.

A–C, F: Right latero-ventral view in different focal planes; D, E: left lateral view. A–C+F and D+E are the same individual.


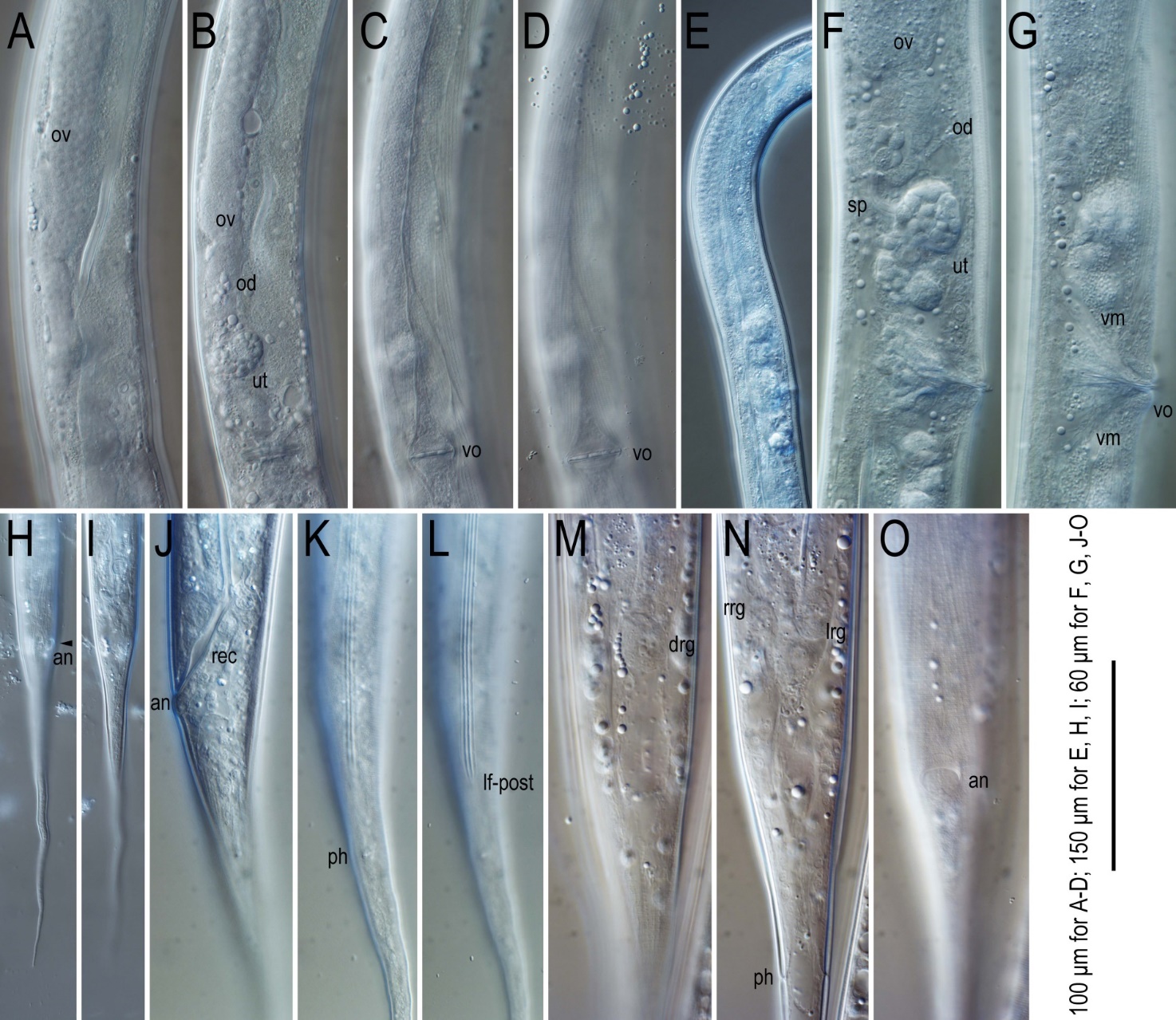


**Figure S6.** Female vulval and anal regions of *Caenorhabditis auriculariae*.

A–D: Ventral view of the anterior gonad region in different focal planes; E: right lateral view of whole anterior gonad; F: close-up of vulval region of E; H, I: right latero-ventral view of the entire tail region in different focal planes; J–L: left lateral view of the anal region in different focal planes; M–O: ventral view of the anal region in different focal planes. In A–D, “ov”, “od”, “ut”, and “vo” indicate the ovary, oviduct, uterus, and vulval opening, respectively. In F and G, “ov”, “od”, “ut”, and “vo” are the same as in A–D, and “sp” and “vm” indicate spermatheca and vulval muscle, respectively. In H-L, “an”, “rec”, “ph”, and “lf-post” indicate anal opening, rectum, phasmid, and posterior end (ending) of the lateral field, respectively. In M–O, “an” and “ph” are the same as in H–L, and “drg”, “rrg”, and “lrg” indicate the dorsal rectal gland, right rectal gland, and left rectal gland, respectively.


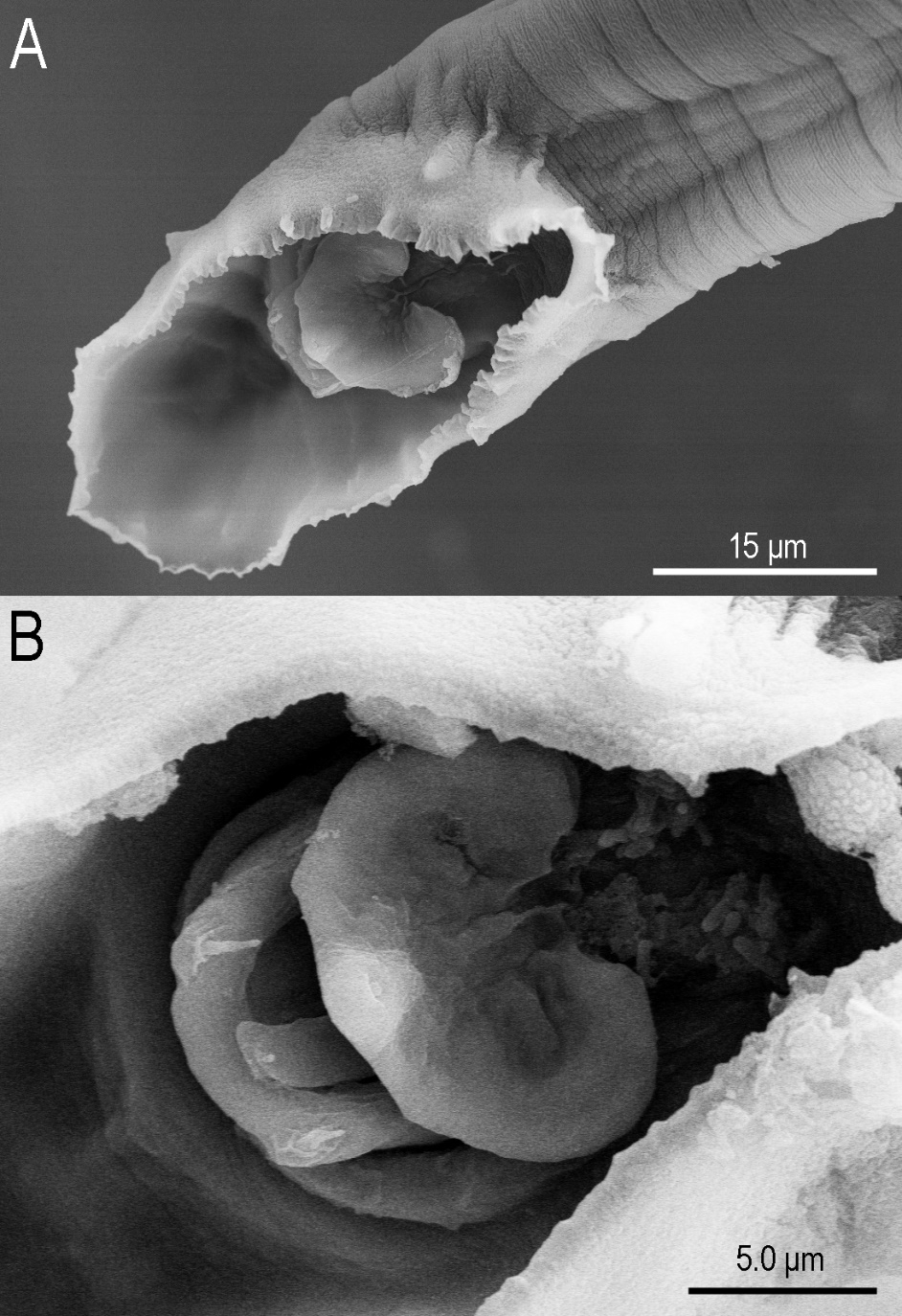


**Figure S7.** Scanning electron micrographs of male tail.

A: Whole tail region in ventral view; B: Cloacal region showing heart-shaped precloacal appendage which was originally described as “bifid cap structure”.
